# 2705. Predictors of Severe COVID-19 among Solid Organ Transplant Recipients

**DOI:** 10.1093/ofid/ofad500.2316

**Published:** 2023-11-27

**Authors:** Marion Gillcrist, Stephen Waller, Ryan M Taylor, Nathan C Bahr, Diane M Cibrik, Zubair Shah, Robert Montgomery, Albert Eid

**Affiliations:** University of Kansas Medical Center, Wichita, Kansas; University of Kansas, Kansas City, Kansas; University of Kansas School of Medicine, Kansas City, Kansas; University of Kansas Medical Center, Wichita, Kansas; University of Kansas Medical Center, Wichita, Kansas; University of Kansas Medical Center, Wichita, Kansas; University of Kansas Medical Center, Wichita, Kansas; University of Kansas Medical Center, Wichita, Kansas

## Abstract

**Background:**

While some studies have found worse COVID-19 outcomes among solid organ transplant (SOT) recipients, others found no difference when comorbidities and other risk factors for severe disease were accounted for. It remains unclear whether immunosuppressive medications increase the risk of progression to severe disease in SOT recipients.

**Methods:**

We conducted a retrospective case-control study of SOT recipients diagnosed with COVID-19 at the University of Kansas Medical Center between January 1, 2020 and December 31, 2022. Severe COVID-19 was defined by hypoxia while the patient was on room air during a 21-day period after the COVID-19 diagnosis was established. We fit a logistic regression model with severe COVID-19 as the response variable and adjusted for prescription of mycophenolate, tacrolimus and prednisone and a set of covariates including age, sex, race, body mass index, hypertension, diabetes mellitus, prespecified time period of COVID-19 diagnosis during the pandemic, chronic heart disease, chronic lung disease, malignancy, end stage renal disease, cirrhosis, smoking, receipt of COVID-19 vaccine, and receipt of monoclonal antibodies.

**Results:**

A total of 570 SOT recipients were diagnosed with COVID-19. Our cohort consisted of 458 kidney, 73 liver, 54 heart, 43 pancreas, and 8 lung recipients. The median patient age was 54 years, 59% were male, and 70% were white. Severe COVID-19 developed in 127/570 (22.3%) patients. A total of 58/570 (10.2%) patients died. A multivariable analysis showed that age (OR = 1.04; p < 0.01), race other than white (OR = 1.88; p = 0.02), chronic heart disease (OR = 1.91, p = 0.01), chronic lung disease (OR = 2.21; p = 0.02), lack of prior COVID-19 vaccination (OR = 2.09; p = 0.01), and not receiving treatment with monoclonal antibodies (OR = 3.82; p < 0.01) were associated with severe COVID-19. Being on treatment with mycophenolate, tacrolimus, or prednisone at the time of COVID-19 diagnosis was not associated with severe disease.

**Conclusion:**

Patients receiving mycophenolate, tacrolimus, or prednisone at the time of COVID-19 diagnosis were not more likely to experience severe disease. Overall mortality in our SOT population was 10%.

**Disclosures:**

**All Authors**: No reported disclosures

